# Computational Study on the Inhibitor Binding Mode and Allosteric Regulation Mechanism in Hepatitis C Virus NS3/4A Protein

**DOI:** 10.1371/journal.pone.0087077

**Published:** 2014-02-25

**Authors:** Weiwei Xue, Ying Yang, Xiaoting Wang, Huanxiang Liu, Xiaojun Yao

**Affiliations:** 1 State Key Laboratory of Applied Organic Chemistry, Department of Chemistry, Lanzhou University, Lanzhou, China; 2 Chinese Academy of Medical Sciences and Peking Union Medical College, Beijing, China; 3 School of Pharmacy, Lanzhou University, Lanzhou, China; 4 State Key Laboratory of Quality Research in Chinese Medicine, Macau Institute for Applied Research in Medicine and Health, Macau University of Science and Technology, Taipa, Macau, China; Wake Forest University, United States of America

## Abstract

HCV NS3/4A protein is an attractive therapeutic target responsible for harboring serine protease and RNA helicase activities during the viral replication. Small molecules binding at the interface between the protease and helicase domains can stabilize the closed conformation of the protein and thus block the catalytic function of HCV NS3/4A protein via an allosteric regulation mechanism. But the detailed mechanism remains elusive. Here, we aimed to provide some insight into the inhibitor binding mode and allosteric regulation mechanism of HCV NS3/4A protein by using computational methods. Four simulation systems were investigated. They include: apo state of HCV NS3/4A protein, HCV NS3/4A protein in complex with an allosteric inhibitor and the truncated form of the above two systems. The molecular dynamics simulation results indicate HCV NS3/4A protein in complex with the allosteric inhibitor 4VA adopts a closed conformation (inactive state), while the truncated apo protein adopts an open conformation (active state). Further residue interaction network analysis suggests the communication of the domain-domain interface play an important role in the transition from closed to open conformation of HCV NS3/4A protein. However, the inhibitor stabilizes the closed conformation through interaction with several key residues from both the protease and helicase domains, including His57, Asp79, Asp81, Asp168, Met485, Cys525 and Asp527, which blocks the information communication between the functional domains interface. Finally, a dynamic model about the allosteric regulation and conformational changes of HCV NS3/4A protein was proposed and could provide fundamental insights into the allosteric mechanism of HCV NS3/4A protein function regulation and design of new potent inhibitors.

## Introduction

**Figure 1:**
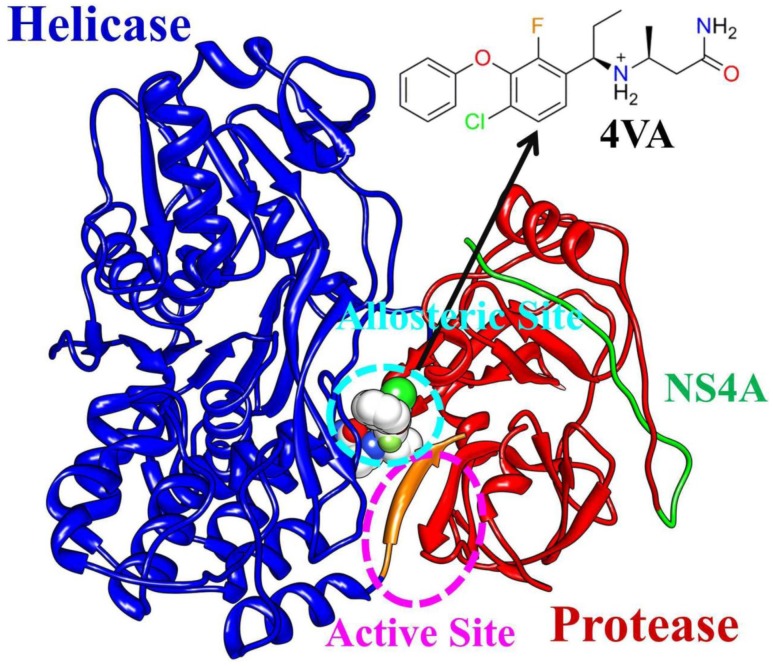
Structural model of HCV NS3/4A protein, including protein domains helicase (blue) and protease (red), cofactor NS4A (green) and the allosteric inhibitor 4VA (gray). The C-terminal β-strand of HCV NS3 helicase domain (amino acids 626–631) is shown in orange.

Hepatitis C virus (HCV) infection is a leading cause of chronic hepatitis, liver cirrhosis, and hepatocellular carcinoma worldwide. It is estimated that a minimum of 3% of the world’s population (180 million people) are affected by HCV [1]. Nonstructural protein 3 (NS3) of HCV, along with the viral NS4A cofactor peptide, is an essential member of HCV replication complex [2]. NS3 protein contains a serine protease and an RNA helicase (Figure 1). The serine protease domain (amino acids 1–180) in the N-terminal performs the cis cleavage to release itself from the polyprotein [3]. The RNA helicase domain (amino acids 181–631) in the C-terminal binds to nucleic acid chains and, fueled by ATP hydrolysis, tracks along them in a 3′ to 5′ direction to displace annealed strands or bound proteins [4]. NS4A cofactor contributes to the proper positioning of the catalytic triad (His57, Asp81, and Ser139) and the substrate.

NS3/4A protein has been proved to be a promising target for developing anti-HCV drugs in recent years. Binding of a ligand at the active site or the allosteric site of HCV NS3/4A can specifically inhibit the protein functional properties. In the past decade, much more attention focused on HCV NS3/4A protease and two drugs, boceprevir [5] and telaprevir [6] were approved by U.S. FDA recently [7]. These two drugs are the first direct-acting antiviral agents (DAAs) against NS3/4A protease and represent a major breakthrough for the treatment of HCV infection. Unfortunately, rapid emergence of drug resistance mutations in HCV NS3/4A protease leads to reduced drug sensitivity to all protease inhibitors [8], [9]. In addition, the shallow substrate binding groove of NS3/4A protease suggested that discovery of a potent, small-molecule, and orally available drug candidate would be an enormously challenging task [10]. Thus, it is urgent to develop new molecules with better efficacy than existing drugs that target NS3/4A protease. Recently, X-ray crystallographic screening of the full length NS3/4A protein leads to the discovery of a novel allosteric binding site [11]. Compared to the active site of NS3/4A protease, current anti-HCV research received little attention on this allosteric site located at the protease-helicase interface of NS3/4A protein. However, the sequence analysis of the allosteric site suggests that the allosteric site residues have a high degree of conservation [11]. Moreover, inhibitors targeting this novel allosteric site show equivalent potency against a number of clinically observed mutant [12], and they were administered in combination with other classes of DAA’s which increases antiviral activity and raise the genetic barrier to drug resistance [13], [14]. That is to say that allosteric inhibition HCV NS3/4A protein activity with small molecules can overcome the drug resistance challenges of targeting the protease active site. Therefore, developing therapeutic agents that directly target and regulate this novel allosteric site may be a dominant pharmacological strategy over classic protease inhibitors, and the study of the allosteric regulation mechanism of HCV NS3/4A protein will be useful for design of new potent inhibitors targeting this site [12].

It is reported that HCV NS3/4A protein have both open (active) and closed (inactive) conformations, and equilibrium exists between an open and closed conformation of the protein [11], [15]. The closed conformation is the product of cis-cleavage (NS3/NS4A), with the C-terminus occupying the protease active site [14], [16]. In the available X-ray crystal structures of apo HCV NS3/4A, the protein adopts the closed, autoinhibited conformation [14]–[16]. However, the stabilization of NS3/4A protein autoinhibited conformation via binding of allosteric inhibitors, such as 4VA studied in this work (Figure 1), could have the ability to inhibit both the helicase and protease enzymatic functions of the protein [11], [12]. Allosteric inhibitor 4VA is different from existing protease inhibitors in their physical properties, nonpeptidic nature and biological profile [11], [12]. In contrast, the open conformation is required for both protease and helicase activities, and this is the conformation which inhibited by the protease inhibitors such as boceprevir and telaprevir [14]–[16]. In addition, studies on apo form of the full-length and the truncated (with the C-terminal β-strand of the protein removed) HCV NS3/4A protein suggested that the C-terminal β-strand of the protein act as a toggle that alters the structural and functional properties of the protein [15]. Unfortunately, the crystal structure of the protein in an open, active conformation has not been determined. Therefore, as for HCV NS3/4A protein, there are still some critical problems needed to solve because the information provided by reported experimental studies are not enough to reveal detailed structural information underlying the allosteric regulation mechanism. Herein, we are interested in the conformational dynamics of the apo HCV NS3/4A protein (full-length and truncated), the binding mode of the inhibitor binds in the novel allosteric site, and the molecular mechanism of the inhibitor to stabilize the closed inactive conformation of the protein.

Computational methods, such as molecular dynamics simulation combined with principal component analysis, clustering analysis, cross-correlation analysis, and residue interaction network analysis can provide significant information from atomic level about the conformational change details and information communication of proteins during the interactions, which can help us understand the allosteric regulation mechanism [17]–[23]. On the basis of molecular dynamics simulation, binding free energy calculation and free energy decomposition analysis are also wildly used to predict the binding affinities of the inhibitor upon protein and to identify the key protein residues responsible for the binding of an inhibitor, which are useful for the structure based drug design of new potent inhibitors [24]–[34].

In the present study, we have utilized a combination of computational techniques to generate an ensemble view of dynamic properties of the allosteric mechanism of HCV NS3/4A protein function regulation. It is found that the fluctuation of the apo HCV NS3/4A protein subdomains (residue 101–171 and 331–420) changed dramatically when compared to the inhibitor bound systems. Further comparative analysis of the dynamic behavior showed that the truncated apo model of NS3/4A protein adopts an open active conformation associated with the protein activity, whereas the inhibitor can inhibit the protein function by stabilizing the closed inactive conformation through binding at the allosteric site. The results from our study can give valuable insights into the allosteric regulation mechanism of HCV NS3/4A protein and provide some useful clues for design of small molecules inhibiting HCV infection by targeting the allosteric site.

## Materials and Methods

### System Setup

Four systems were used for the simulation of the underlying dynamics and allosteric regulation mechanism of HCV NS3/4A protein function. The details about the studied systems are listed in Table 1. The simulations of the X-ray crystal structure of 1CU1 [16] and 4B73 [11] were designed to prove the importance of the stability of an autoinhibited form of HCV NS3/4A protein by a small molecular binding at the novel allosteric site. As shown in Table 1, the truncated forms of HCV NS3/4A protein models were created by *in silico* removing the six C-terminal residues coordinates starting from above structures 1CU1 and 4B73. The truncated apo system was built to get a extend conformation which has shown to be essential for both protease and helicase function [15], [16]. The inhibitor bound truncated model was designed to investigate whether the conformational changes from inactive to active form is solely caused by the presence of inhibitor in the allosteric site or not.

**Table 1:** Summary of the simulation systems.

systems	simulation time	starting structure
apo protein	100 ns	X-ray structure of HCV NS3/4A protein (PDB ID code 1CU1)
inhibitor bound protein	100 ns	X-ray structure of HCV NS3/4A protein complexed with inhibitor (PDB ID code 4B73)
apo protein (truncated)	100 ns	removal of the six C-terminal residues from apo protein
inhibitor bound protein(truncated)	100 ns	removal of the six C-terminal residues from protein complexed with inhibitor

All the structures were then modeled by using the program LEaP embedded in AMBER10 [35] with the standard AMBER03 force field [36] for protein, including adding all missing hydrogen atoms of the protein. The force field parameters for the inhibitor were described by the General AMBER Force Field (GAFF) [37] and Restrained Electrostatic Potential (RESP) [38]–[40] partial charges. Geometry optimization and the electrostatic potential calculations were performed at the HF/6-31G* level of Gaussian09 suite [41]. Sodium ions are added to neutralize the overall charge of the system. Each system was solvated in a rectangular pre-equilibrated box of TIP3P [42] water molecules. Sufficient solvent was added to provide a minimum distance of 8 Å between any protein atom and the edge of the box. This yielded a simulation box containing about 20000 water molecules with initial dimensions of 82 Å × 93 Å × 100 Å.

### Molecular Dynamics Simulation

All molecular dynamics simulations were carried out using the AMBER 10 with periodic boundary condition. The prepared systems were subjected to initial energy minimization by two steps, applying harmonic restraints with a force constant of 500.0 kcal/(mol·Å^2^) to all protein atoms and allowing all atoms to move freely in turn. In each step, energy minimization was performed by the steepest descent method for the first 3000 steps and the conjugated gradient method for the subsequent 2000 steps. Thereafter systems were sequentially heated up from 0 to 310.0 K over 100 ps in the *NVT* ensemble and equilibrating to adjust the solvent density under 1 atm pressure over 50 ps in the *NPT* ensemble simulation by restraining all atoms of the structures with a harmonic restraint weight of 10.0 kcal/(mol·Å^2^). An additional three molecular dynamics equilibrations of 50 ps each were performed with the decreased restraint weights of 5.0, to 1.0, to 0.1 kcal/(mol·Å^2^), respectively. These were followed by the last molecular dynamics equilibration step of 50 ps by releasing all the restraints. Afterward, production molecular dynamics simulations were carried out without any restraint on these four systems in the *NPT* ensemble at a temperature of 310.0 K and a pressure of 1 atm. During the simulations, periodic boundary conditions were employed and direct space interactions were truncated at a distance 10 Å with long range contributions from the electrostatics included using the particle mesh Ewald (PME) method [43]. Van der Waals contributions beyond the cutoff were included via the use of an isotropic long range correction. The SHAKE algorithm [44] was used to restrain bond lengths involving bonds to hydrogen atoms. An integration step of 2 fs was used and the coordinates of the trajectories were saved every 1 ps.

### Principal Component Analysis

Principal component analysis was carried out using the PTRAJ module of AmberTools 1.2 suite to each of the trajectory from molecular dynamics simulations. Prior to performing PCA, 5000 snapshots (0 ns to 100 ns) or 1000 (80 ns to 100 ns) snapshots were taken every 20 ps for each of the simulation trajectory, and overall translation or rotation motion were removed by fitting the coordinate data to the average structure to obtain the proper trajectory matrix. The standardized trajectory data is then utilized to generate a covariance matrix between the Cα atoms *i* and *j*, which are defined as:
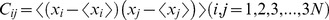
(1)where *x_i_* and *x_j_* are Cartesian coordinates of the *i*th and *j*th Cα atom, N is the number of the Cα atoms considered, and <*x_i_*> and <*x_j_*> represent the time average over all the configurations obtained in molecular dynamics simulation [45], [46].

### Clustering Analysis

In order to identify the most significant conformational states, the conformations sampled during the molecular dynamics simulation excluding the equilibration periods are clustered by k-means clustering using the kclust module of Multiscale Modeling Tools for Structural Biology (MMTSB) Tool Set [47] having RMSD of the Cα atoms as the similarity measure.

### Dynamical Cross-correlation maps analysis

The correlation matrix was calculated across all Cα atoms of HCV NS3/4A protein. The elements of this matrix, *s_ij_*, assign a value between −1.0 and 1.0 that indicates the degree to which the fluctuations of atom *i* are correlated with those of atom *j* over the course of the simulation trajectory according to the following equation:
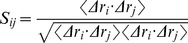
(2)where Δ*r_i_* and Δ*r_j_* are the displacement vectors for atoms *i* and *j*, and the <…> denotes trajectory averages [48].

### Thermodynamic Calculation

The binding free energy (ΔGbind) for the inhibitor association to HCV NS3/4A protein was calculated by using Molecular Mechanics/Poisson-Boltzmann Surface Area (MM/PBSA) methods [49] with 1000 snapshots at 10 ps interval from the last equilibrated 10 ns trajectory as follows:
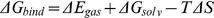
(3)where Δ*E_gas_* is the change in the sum of the bonded (internal), and nonbonded electrostatic (Δ*E_ele_*) and van der Waals energies (Δ*E_vdW_*) between the unbound states and the bound state. These energy contributions are computed from the atomic coordinates of the protein, ligand and complex using the molecular mechanics energy function. Change in the solvation free energy term (Δ*G_solv_*) contains both polar (Δ*G_PB_*) and nonpolar (Δ*G_SA_*) contributions. The polar contributions were calculated by solving the PB equation, with dielectric constants for solute and solvent set to 1 and 80, respectively [50]. The nonpolar contributions were estimated by the solvent-accessible surface area (SASA) determined using a water probe radius of 1.4 Å and the surface tension constant *γ* was set to 0.0072 kcal/(mol/Å^2^) [51]. −*T*Δ*S* is the solute entropy change, which can be evaluated via normal mode analysis [52]. Normal mode analysis was carried out in the AMBER10 NMODE module by performing a conjugate gradient minimization. Because this analysis requires extensive computer time, 100 snapshots extracted from the last equilibrated 10 ns trajectory of the simulation with 100 ps time intervals were used to estimate the order of magnitude of the solute entropy. Each snapshot was fully minimized with a distance dependent dielectric function 4R_ij_ (the distance between two atoms) until the root mean square of the elements of the gradient vector was less than 1×10^−4^ kcal/(mol·Å).

In order to obtain the contributions of individual residue to the total binding free energy, we perform free energy decomposition in a per-residue basis by using the MM/GBSA decomposition program [53] in the AMBER 10 without consideration of the contribution of entropy by:
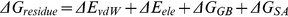
(4)where Δ*E_vdW_* and Δ*E_ele_* are non-bonded van der Waals interactions and electrostatic interactions between the inhibitor and each residue in the gas phase. Δ*G_GB_* and Δ*G_SA_* are the polar and nonpolar contributions to the inhibitor-residue interaction. All energy components in Eq. (4) were calculated using the 1000 snapshots taken from the last equilibrated 10 ns simulation.

### Residue Interaction Network Analysis

The representative protein structure derived from molecular dynamics simulation was used for residue interaction network analysis. To derive the physic-chemically valid protein residue interaction network residue interaction network, the web server RING (http://protein.bio.unipd.it/ring/complex.html) was used. In contrast to define residue interactions on the basis of spatial atomic distance between residues, RING distinguishes different residue interaction types and quantifies the strength of individual interactions. To this end, RING relies on REDUCE [54] to correct the orientation errors of important chemical groups in the side chain of certain amino acids and calculate the coordinates hydrogen atoms of the 3D protein structure. The resulting structural model was used in PROBE [55], in order to identify noncovalent interaction residues between the atoms of each pair of considered residues. Two residues are defined as in contact if any two of their atoms exist at least one van der Waals interaction. The network generated from the 3D structure was used to visualize the network by Cytoscape [56] and the plugin RINalyzer [57]. In a network, the nodes represent the protein residues and the edges between them represent the noncovalent interactions. The edges are labeled with an interaction type, usually including interatomic contact, hydrogen bond, salt bridge and so on. Using the NetworkAnalyzer [58] plugin of Cytoscape, we performed the protein topological parameters analysis of shortest path betweenness and closeness centrality. The betweenness and closeness centrality of each node is a number between 0 and 1 [59]. Network analysis of protein structures has shown that amino acid with high shortest path betweenness values involved in stabilizing the protein structures and the residues with high closeness values are likely to be functionally important [60]–[62]. In addition, communication within protein residues is crucial for the biological functioning of the protein [63], and closeness centrality is a measure of how quickly information spreads from a given node to other reachable nodes in the network [64].

## Results and Discussion

### Structure and Dynamics of HCV NS3/4A Protein

**Figure 2:**
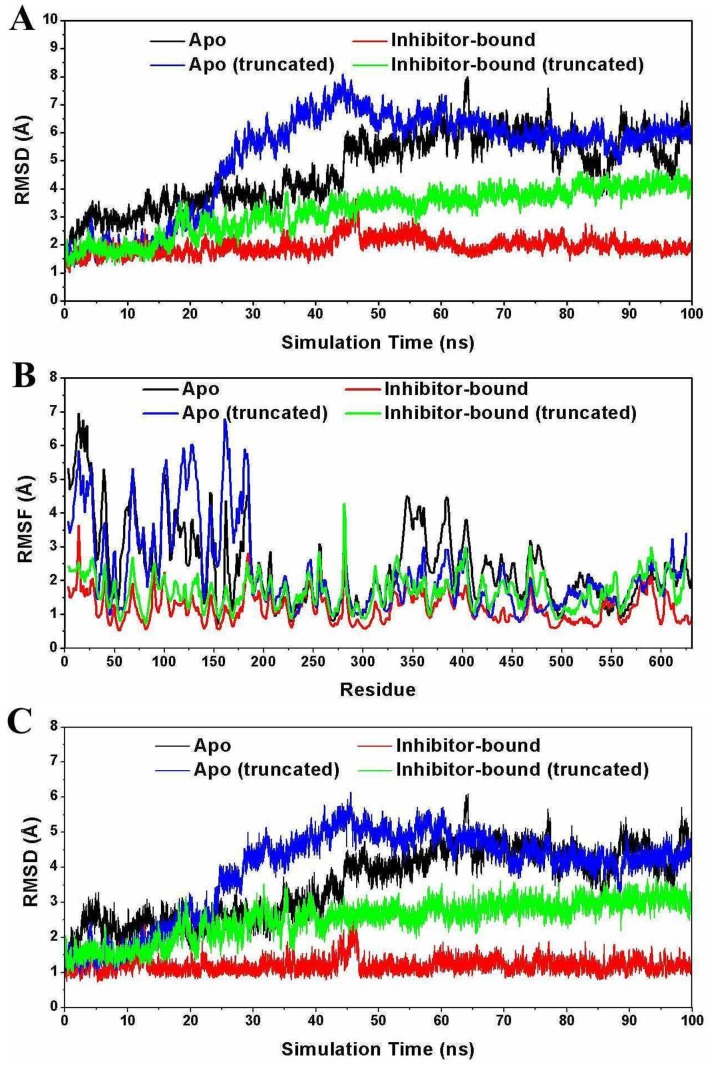
Global properties for molecular dynamics simulations. (A) The backbone atom root mean square deviation (RMSD) for apo, inhibitor bound, apo (truncated) and inhibitor bound (truncated) HCV NS3/4A protein, calculated with respect to the initial structure during the 100 ns molecular dynamics simulation. (B) Root mean square fluctuations (RMSF) of Cα for apo, inhibitor bound, apo (truncated) and inhibitor bound (truncated) HCV NS3/4A protein averaged over the simulation. (C) RMSD of the backbone atoms of residues 101–172 and 331–420 with respect to the first snapshot as a function of time.

The X-ray structure of apo HCV NS3/4A protein revealed that the C-terminal β-strand of helicase domain occupies the protease active site and the protein is in an autoinhibited inactive state [16]. Ligands binding at the allosteric site can stabilize the autoinhibited conformation (closed) of the protein [16]. The allosteric inhibitor 4VA shown in Figure 1 is a representative member for these ligands and was used as a model molecule for its high affinity to study the functional role of the allosteric site [11]. Herein, to elucidate the inhibition mechanism of 4VA bound in this allosteric site, 100 ns molecular dynamics simulation were conducted respectively on both the X-ray structures of inactive apo and inhibitor bound HCV NS3/4A protein. First, by comparing the stable fluctuation evolution of the root-mean-square deviation (RMSD) values of all backbone atoms relative to the starting structures (Figure 2A), we observed that large conformational change occured in the apo HCV NS3/4A protein, while the inhibitor bound system remained relatively stable throughout the simulation. Then, analysis of the root-mean-square fluctuations (RMSF) and RMSD of the subdomains (residue 101–171 and 331–420), shown in Figure 2B and 2C, revealed that the conformation of these two regions changed dramatically, as in the apo structure.

**Figure 3:**
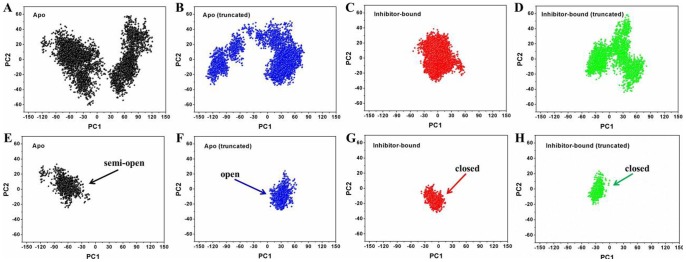
Principal components analysis to the Cα atom motions of the simulation. (A–D) The cloud represents the four 100 ns trajectories projected onto the first two eigenvectors. (E–H) The cloud represents the four equilibrium 20 ns trajectories projected onto the first two eigenvectors. The clouds colored in black, red, blue and, green display apo, inhibitor bound, apo (truncated) and inhibitor bound (truncated) HCV NS3/4A protein, respectively.

To better characterize the nature of collective motions, principal component analysis were carried out on the simulation trajectories. Principal component analysis studies focus on these dominant motions and Figure 3 shows the projection of each member of the ensemble onto the plane defined by the top two eigenvectors. Principal component analysis of the whole 100 ns trajectory for each system indicated that the apo system and its truncated form covered the larger region of the phase space than that of the inhibitor bound systems (Figures 3A–3D). Additionally, we performed principal component analysis on the equilibrium trajectory (80 ns to 100 ns) for each system and shown in Figures 3E–3H. As can be seen from Figures 3E–3H, the projections of the inhibitor bound, apo, and truncated apo models cover the different regions of the phase space. It is demonstrated that after convergence, the simulated systems are formed in different conformations.

Clustering analysis on the whole 100 ns trajectory for each system indicates that there are mainly two conformations for the apo (Figures S1A and S1B, PDB S1 and S2) and truncated apo (Figures S1D and S1E, PDB S3 and S4) systems in the simulation. One is the closed conformation (Figures S1A and S1D, PDB S1 and S3), and the other one is the intermediate conformation for apo system (Figure S1B, PDB S2) or the open conformation for the truncated apo system (Figure S1E, PDB S4). However, only one conformation (closed) was found for the inhibitor bound system (Figure S1C, PDB S5) and the inhibitor bound truncated system (Figure S1F, PDB S6). However, it is implied that the inhibitor binding plays a critical role in stabilizing HCV NS3/4A protein closed conformation.

**Figure 4:**
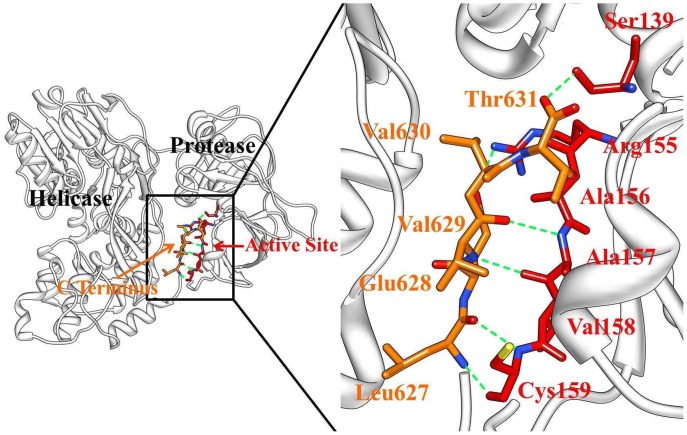
Hydrogen bond network between the C-terminal β-strand of helicase and protease active site in HCV NS3/4A protein (apo). The protease and helicase domains are shown in cartoon and colored in gray. The six C-terminal residues of the helicase and the protease active site residues are represented by orange and red sticks, respectively. Green dashed lines represent the hydrogen bond.

Additionally, it is reported that the key backbone hydrogen bond network between the six C-terminal residues of the helicase domain and active site of the protease domain (Figure 4) is important to stabilize the protein in a closed conformation [11], [15], [16]. In this study, the presences of these hydrogen bond interactions were examined along the time of each molecular dynamics simulation. The persistence of identified hydrogen bond was summarized in Table 2. After the comprehensive comparison of the hydrogen bond distance, angle, as well as the occupancy, we found that three of the six hydrogen bonds are weakened in the apo system.

**Table 2:** Analysis of hydrogen bond network between HCV NS3/4A helicase and protease domains.

hydrogen bonds network^a^		distance(Å)		angle(°)		occupancy(%)^b^	
doner	acceptor	apo	bound	apo	bound	apo	bound
Ala157 (O)	Val629 (N-H)	2.80	2.79	165.20	165.73	95.24	96.80
Leu627 (O)	Cys159 (N-H)	2.85	2.86	163.63	164.68	70.14	73.71
Val629 (O)	Ala157 (N-H)	2.89	2.89	158.89	152.85	53.31	51.09
Thr631 (O)	Ser139 (OG-HG)	2.59	2.60	165.66	163.16	38.30	50.32
Cys159 (O)	Leu627 (N-H)	2.89	2.89	156.59	162.38	14.49	51.57
Glu628 (OE1)	Arg155 (NH2-HH22)	2.83	2.84	154.44	161.41	5.10	45.58

a The hydrogen bonds are determined by the acceptor···donor atom distance of less than 3.5 Å and acceptor···H-donor angle of greater than 120°.

b Occupancy(%): to evaluate the stability and the strength of the hydrogen bond.

### Structure and Dynamics of the Truncated HCV NS3/4A Protein

Large scale conformational changes occur by analyzing the simulation trajectory obtained from the truncated apo system. The dynamical response of the truncated apo HCV NS3/4A protein was first evaluated by computing the RMSF and RMSD values (Figure 2B and 2C), indicating the subdomain motions including the residues 101–171 and 331–420. Then, principal component analysis (Figure 3B) and clustering analysis (Figure S1E, PDB S4) demonstrate that after the relatively long time simulation, the truncated apo structure reached the final open conformation. However, this structural rearrangement is promoted by the removing the hydrogen bond interactions between the C-terminal β-strand residues of the helicase domain and the protease active site residues (Figure S2). Therefore, the C-terminal β-strand of HCV NS3/4A was suggested to act as a toggle in altering the protein to adopt different conformational states.

**Figure 5:**
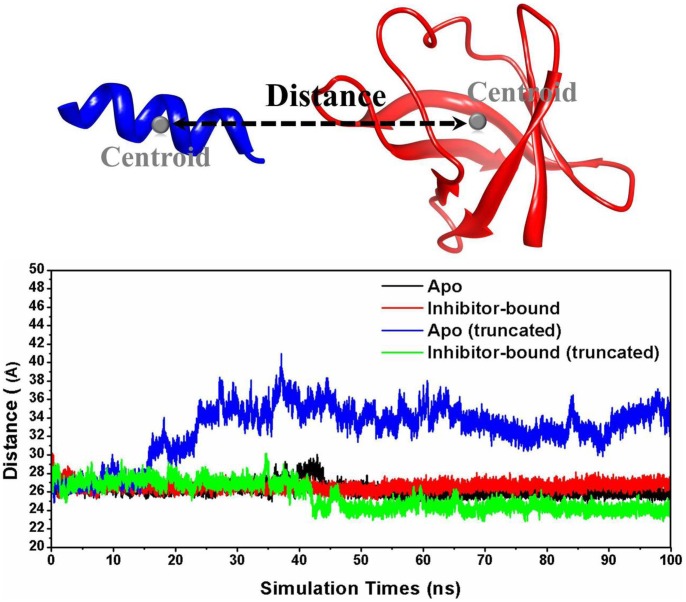
Distance between the backbone centers of mass of the helicase residues 614–625 and protease residues 103–171, as it varies during apo, inhibitor bound, apo (truncated) and inhibitor bound (truncated) HCV NS3/4A protein simulation.

In order to monitor the opening progress of HCV NS3/4A protein, the distance between the backbone centers of mass of the helicase residues 614–625 and protease residues 103–171 during the simulations were calculated and shown in Figure 5. As seen in Figure 5, the truncated protein undergoes a domain movement that populates mainly the open conformation. It is noted that even in the case of truncated system, the allosteric inhibitor still allows the protein to be in its inactive form. This further indicates that the allosteric inhibitor is an important factor for the conformational stabilization of the closed state.

### Analysis of the Inhibitor Binding at the Interface of HCV NS3/4A Protease and Helicase Domains

**Figure 6:**
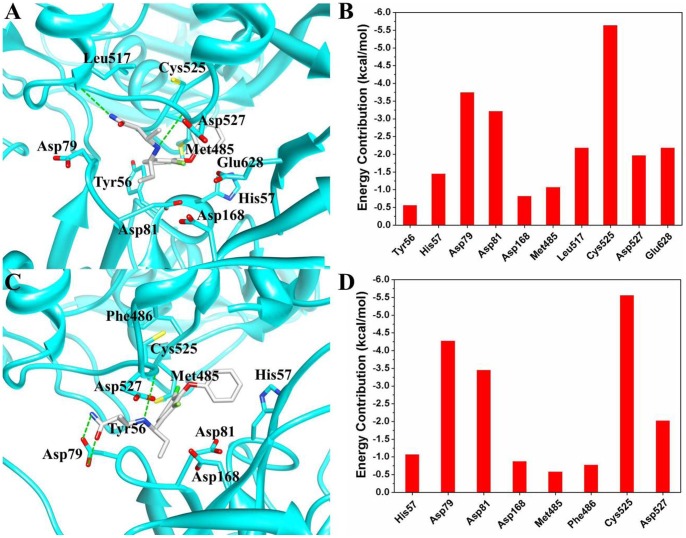
Structural models and per-residue interaction spectrums of the residues of (A–B) HCV NS3/4A protein and (C–D) its truncated protein with the allosteric inhibitor. The representative structures extracted from the simulation trajectories were used. The proteins are shown in cyan cartoon. The inhibitor is shown in gray stick model. The residues with an absolute value larger than 0.5/mol for the inhibitor-residue interactions are shown.

**Figure 7:**
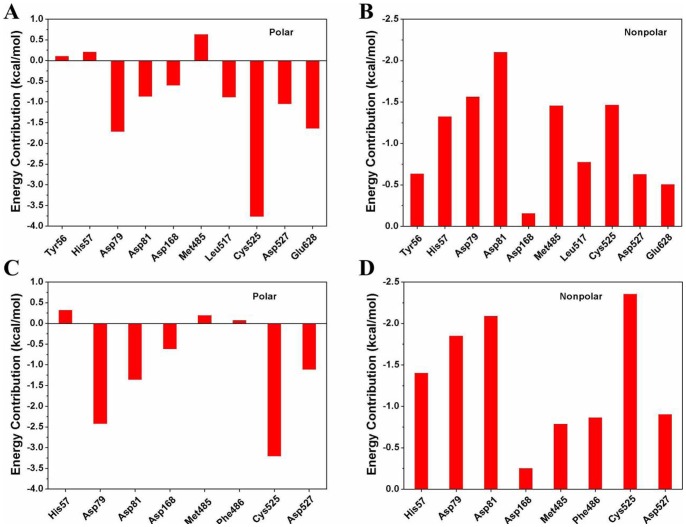
The polar and nonpolar interaction energies between HCV NS3/4A protein (A–B) and its truncated protein residues (C–D) with the allosteric inhibitor.

In this section, we focused our analysis on the binding properties of the inhibitor to the newly identified allosteric pocket of HCV NS3/4A protein. The pocket is located near, yet is distinct from, the protease active site, which is occupied by the C terminus of the helicase domain in the crystal structure (Figure 1). Depicted in Figures 6A and 6C are the representative structures of the simulated conformational ensemble of the inhibitor 4VA binding to the different models. Although the affinity of the inhibitor 4VA to the truncated protein yet has not been determined, however, in order to compare the binding free energy difference of the inhibitor in two models, we calculated the binding free energy by using the MM/PBSA approach and the results are collected in Table 3. The free energy component given in Table 3 suggested that the majority of the favorable contributions for the 4VA binding are Δ*E_vdW_* and Δ*E_ele_*, whereas the Δ*G_solv_* (Δ*G_solv_* = Δ*G_PB_* +Δ*G_SA_*) and −*T*Δ*S* create the unfavorable contribution to the binding energy. In order to provide a more precise quantitative interpretation of binding affinity, binding free energy decomposition analysis were performed to determine the individual enthalpic contribution to the interaction energy between inhibitor and HCV NS3/4A protein residue pairs. As shown in Figures 6A and 6B, the inhibitor 4VA binds with HCV NS3/4A protein mainly through interactions with residues include Tyr56, His57, Asp79, Asp81, Asp168, Met485, Leu517, Cys525, Asp527, and Glu628. Among these interactions, inhibitor forms hydrogen bond interactions with backbone atoms of residues Leu517 and Cys525. Figure 7 further gives the decomposition of the binding free energy into the contributions of the polar and nonpolar interactions for the favorable residues. According to Figures 7A and 7B, the main favorable binding forces of residues Tyr56 and His57 are the nonpolar contributions, while the polar contributions are unfavorable. For the other residues, both of the polar and nonpolar contributions are favorable for binding, but the polar contributions are more favorable for Asp79, Leu517, and Cys525 (Figures 7C and 7D).

**Table 3:** Binding free energy and the contributions of for the inhibitor in HCV NS3/4A protein^a^.

systems	contributions							
	Δ*E_ele_*	Δ*E_vdW_*	Δ*G_PB_*	Δ*G_SA_*	Δ*H_bind_*	−*T*Δ*S*	Δ*Gcal bind*	Δ*Gexp bind* b
inhibitor bound protein	−180.15±0.29	−48.67±0.08	200.00±0.28	−3.67±0.002	−32.49±0.12	16.76±0.48	−15.73	−10.08
inhibitor bound protein (truncated)	−123.66±0.38	−41.28±0.08	142.27±0.34	−3.61±0.004	−26.28±0.14	17.46±0.52	−8.82	

a All energies are in kcal/mol.

b The experimental value Δ*Gexe bind* was derived from the experimental IC_50_ value in reference by using the equation Δ*Gexe bind* ≈ *RT*ln(IC_50_) at 310.0 K.

The predicted free energies (Δ*Gcal bind*) of the 4VA bound to the full-length and truncated HCV NS3/4A protein are −15.73 and −8.82 kcal/mol. Comparison of their binding modes shown in Figures 6A and 6C indicated the removing of the C-terminal residues affects the location of the inhibitor. It is supposed that hydrogen bond is essential in stabilizing protein-ligand interaction mode. However, the flipped orientation of the inhibitor in Figure 6C lost the important hydrogen bond interaction with Leu517 but form hydrogen bond interaction with Asp79, which is consistent with the contributions of the polar energy data shown in Figure 7.

### Community Network between HCV NS3/4A Protease and Helicase Interface

**Figure 8:**
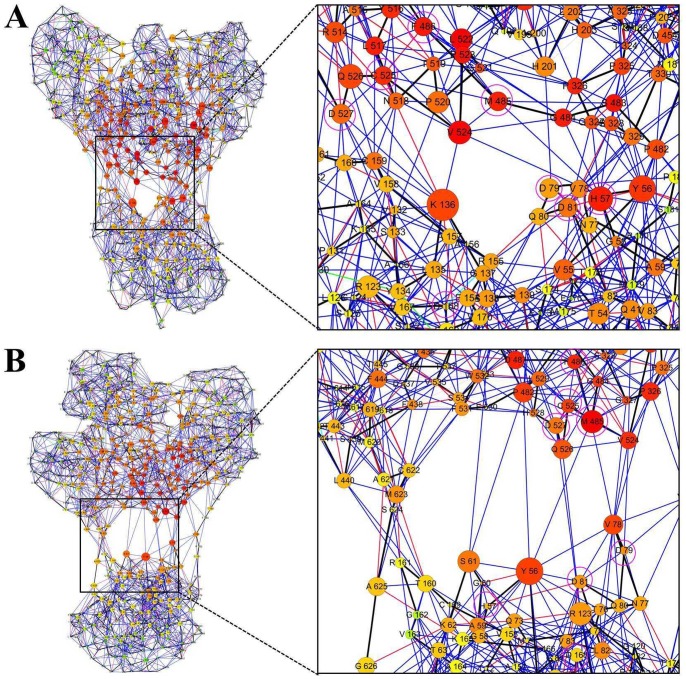
Protein residue interaction network and its communities of the (A) apo (truncated) and (B) inhibitor bound HCV NS3/4A protein. Different views of the corresponding RIN are displayed. The edges are colored with respect to their interaction type: backbone (black); interaction between closest atoms (blue); hydrogen bond (red); salt bridge (cyan); π-cation interaction (green); π-π interaction (gray). Closeness centrality denoted by nodes color (high values to bright colors). Betweenness centrality denoted by nodes size (high values to large sizes).

It is revealed that the simulation results of the truncated apo (Figure S1E, PDB S4) and the inhibitor bound (Figure S1C, PDB S5) HCV NS3/4A protein represent the active and inactive states of the protein. To better understand the molecular origin of these changes in motion of these two models, the residue interaction network analysis of the protein was used to illustrate the interactions of the protease-helicase interface. As shown in Figure 8, the simulated two structures were transformed into 2D representations of residue interaction network by identifying all interactions between the amino acids. In the network, different types of simultaneous noncovalent residue interactions (edges), that is, interaction between closest atoms, hydrogen bond, salt bridge, π-cation interaction and π-π interaction are considered. Here, we further computed the shortest path betweenness and closeness centrality of each node in the network of the inhibitor bound and the truncated apo HCV NS3/4A protein. It is remarkable that the residues located at the interface between the protease and helicase domains have a relative high value of the closeness centrality. Table 4 shows the comparison of the key residues shortest path betweenness and closeness centrality in the inhibitor bound and the truncated apo HCV NS3/4A protein network. As seen in Table 4, residues His57, Asp79, Asp81, Phe486, Cys525, and Asp527 in truncated apo HCV NS3/4A protein possess higher value of the closeness centrality than that in the inhibitor bound protein. Thus, it is evident that, the communication of the domain-domain interface plays an important role in the open process of HCV NS3/4A protein. However, the inhibitor binding at the allosteric site of HCV NS3/4A protein through interacting with residues His57, Asp79, Asp81, Asp168, Met485, Phe486, Cys525, and Asp527 at the domain-domain interface. Hence, we proposed that binding of the inhibitor at the allosteric site block the information communication between the functional domains.

**Table 4:** Summary of the shortest path betweenness and closeness centrality of selected residues in the network of HCV NS3/4A protein.

residues	apo protein (truncated)		inhibitor bound protein	
	betweenness	closeness	betweenness	closeness
His57	0.21	0.052	0.18	0.0061
Asp79	0.19	0.029	0.19	0.0075
Asp81	0.20	0.026	0.18	0.011
Asp168	0.18	0.0054	0.18	0.011
Met485	0.22	0.026	0.22	0.082
Phe486	0.22	0.025	0.21	0.018
Cys525	0.21	0.024	0.20	0.014
Asp527	0.20	0.033	0.19	0.029

### Conformational Change Reveals the Allosteric Regulation Mechanism in HCV NS3/4A Protein

**Figure 9:**
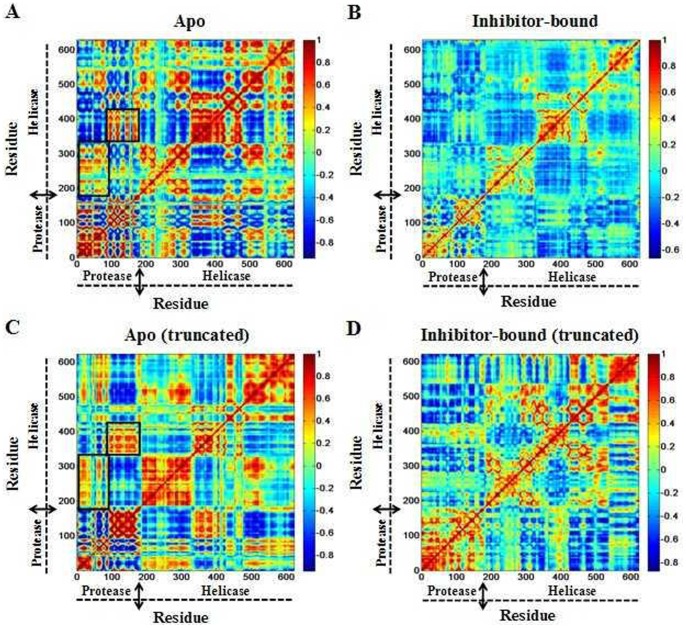
Dynamical cross-correlation maps illustrating the correlation of motion between residues in (A) apo, (B) inhibitor bound, (C) apo (truncated) and (D) inhibitor bound (truncated) HCV NS3/4A protein. The color scale is represented on the right ranging from red to blue: highly positive correlations are in red, highly negative correlations are in blue.

**Figure 10:**
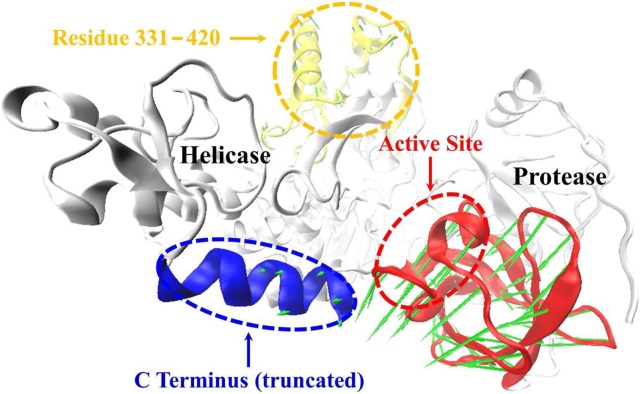
Porcupine plots of the eigenvectors for simulation of apo HCV NS3/4A protein (truncated). The model is shown as a backbone trace. The arrows attached to each backbone atom indicate the direction of the eigenvector and the size of each arrow shows the magnitude of the corresponding eigenvalue. Regions of HCV NS3/4A helicase residues 614–625 and protease residues 103–171 involved in domain movement are highlighted with blue and red, respectively.

The present results demonstrated that the truncated apo form of HCV NS3/4A protein shifts the domains dynamics toward an open conformation (Figure S1E, PDB S4). This phenomenon was significantly related with the high-frequency RMSF and RMSD values (Figures 2B and 2C) which show the interdomain motions observed along simulations of the apo and its truncated models. To gain further insight into the conformational changes, we here investigated the correlation between the motions of residues by performing dynamical cross-correlation maps analysis. Figure 9 shows our results for correlation coefficients of apo and inhibitor bound systems. These coefficients provide information about the correlation between the fluctuations of the positions of the residues. In the case of truncated apo protein, large values are obtained for highly correlated motions (Figure 9C), including residue 101–171 in the protease domain and residue 331–420 in the helicase domain. Porcupine plot shown in Figure 10 is used to display this concerted protein motion. The size of the vectors indicates the extent to which a residue’s motion is correlated with the opening of the conformation. Besides, residues 101–171 and 331–420 in apo protein and residues 1–100 and 200–330 in both apo and its truncated protein also show relatively high correlations (Figures 9A and 9C). Thus, we proposed that the correlated motions of the amino acid residues between HCV NS3/4A functional domains are required for the activity of both the helicase and protease.

In comparison to the apo and its truncated states, the correlated motions in inhibitor bound systems reveal a rapid reduction of correlations, induced by the inhibitor binding (Figures 9B and 9D). Particularly, the largest reduction of correlations is observed for residues 101–171 and 331–420 in the truncated HCV NS3/4A apo protein. This also indicates that the binding of the allosteric inhibitor at HCV NS3/4A protease-helicase domain interface is able to shift the equilibrium toward the closed (inactive) state.

**Figure 11:**
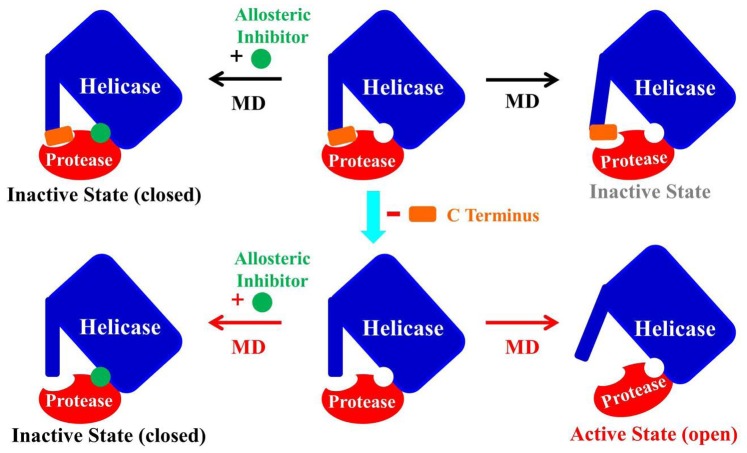
The proposed allosteric mechanism for the regulation of HCV NS3/4A protein function. The helicase domain and the six C-terminal residues of the helicase domain, and the protease domain are represented by boxes and oval. They were colored in blue, red and orange, respectively. The allosteric inhibitor is shown as green round. The arrows in black show the C terminus of the helicase domain occupies the protease active site with and without an inhibitor binding at the allosteric site, stabilizing the protein in a closed conformation. Reactions shown with red arrows are the processes we found in this study. We found that HCV NS3/4A protein possess an extended conformation with the C-terminal residues 626–631 of the helicase domain removed and without the inhibitor binding. And with the inhibitor binding at the allosteric site, it can stabilize the closed conformation of the protein in an inactive state.

On the basis of these results, we have generated an ensemble view of the conformational dynamics and shown the proposed course of allosteric regulation mechanism in Figure 11. The proposed allosteric regulation mechanism shown in Figure 11 indicates that the protein adopts the closed inactive conformation when allosteric inhibitor binding at the protease helicase interface, this can explain the experiment results that small molecules targeting HCV NS3/4A protein in its closed conformation has the potential to inhibit the protein’s helicase and protease activities [11], [12]. The apo structure, especially for its truncated form, reached an open active conformation during the simulation, which is consistence well with the previous observation that C-terminal β-strand of the protein act as a toggle that alters the structural and functional properties of the protein and the protein populates an extended conformation when in its functionally active state [15]. In addition, the conformational changes during the simulation can also help us understand the experimental phenomena that both the open and closed conformations of HCV NS3/4A protein exist when in functional analysis [11].

## Conclusions

In this work, we carried out molecular dynamics simulation to study the inhibitor binding mode and allosteric regulation mechanism in hepatitis C virus NS3/4A. First, by comparing the conformational changes of the constructed four models during the simulation, we found that, in the truncated apo system, the protein adopt the open conformation. The open conformation of the truncated apo structure represents the active state of the HCV NS3/4A protein. However, for the inhibitor 4VA bound HCV NS3/4A protein, even in its truncated form, the allosteric inhibitor allows the protein to be in its closed inactive state. Then, correlation and clustering analysis show evidence of the protein subdomains motions that are crucial for HCV NS3/4A protein activity. Furthermore, residue interaction network analysis of the protein implies that information communication between the residues located at protease-helicase interface may play an important role in the conformational change from closed to open state. In contrast, the allosteric inhibitor binding at the protease-helicase interface through interacting with key residues, such as His57, Asp79, Asp81, Asp168, Met485, Phe486, Cys525, and Asp527, is able to stabilize the closed conformation by blocking the information communication between the two functional domains. These findings will significantly facilitate our understanding of the conformational dynamics in the course of HCV NS3/4A protein allosteric regulation, and the inhibitor that stabilize the closed conformation represent drug candidate alternatives to HCV NS3/4A protease inhibitors.

## Supporting Information

Figure S1 **Clustering analysis of HCV NS3/4A protein motions in different models.** A–F are the representative structural conformations of generated clusters for apo, inhibitor-bound, apo (truncated), and inhibitor-bound (truncated) HCV NS3/4A protein during the 100 ns of molecular dynamics simulation. The regions of protein are individually colored: the helicase residues 614–625 are shown in blue, the C-terminal portion (amino acids 626–631) of the helicase domain orange, the protease residues 103–171 red, the inhibitor green, and the others protein gray.
(TIF)

Figure S2 **The aligned apo and its truncated form of HCV NS3/4A protein structures.** The regions of protein are individually colored: the helicase residues 614–625, the C-terminal portion (amino acids 626–631) of the helicase domain, and the protease residues 103–171 of the apo structure are shown in red, the helicase residues 614–625 and the protease residues 103–171 of the truncated apo structure are shown in blue, and the others protein gray.
(TIF)

PDB S1 **The coordinates of the apo protein representative conformation 1 of generated clusters for the simulation system.**
(PDB)

PDB S2 **The coordinates of the apo protein representative conformation 2 of generated clusters for the simulation system.**
(PDB)

PDB S3 **The coordinates of the apo (truncated) protein representative conformation 1 of generated clusters for the simulation system.**
(PDB)

PDB S4 **The coordinates of the apo (truncated) protein representative conformation 2 of generated clusters for the simulation system.**
(PDB)

PDB S5 **The coordinates of the inhibitor-bound protein representative conformation of generated clusters for the simulation system.**
(PDB)

PDB S6 **The coordinates of the inhibitor-bound (truncated) protein representative conformation of generated clusters for the simulation system.**
(PDB)
