# Supplementary figures and images for: Computational Study on the Inhibitor Binding Mode and Allosteric Regulation Mechanism in Hepatitis C Virus NS3/4A Protein
Source: PLoS One. 2014 Feb 25;9(2):e87077. doi: 10.1371/journal.pone.0087077 (PMC3934852; doi:10.1371/journal.pone.0087077)

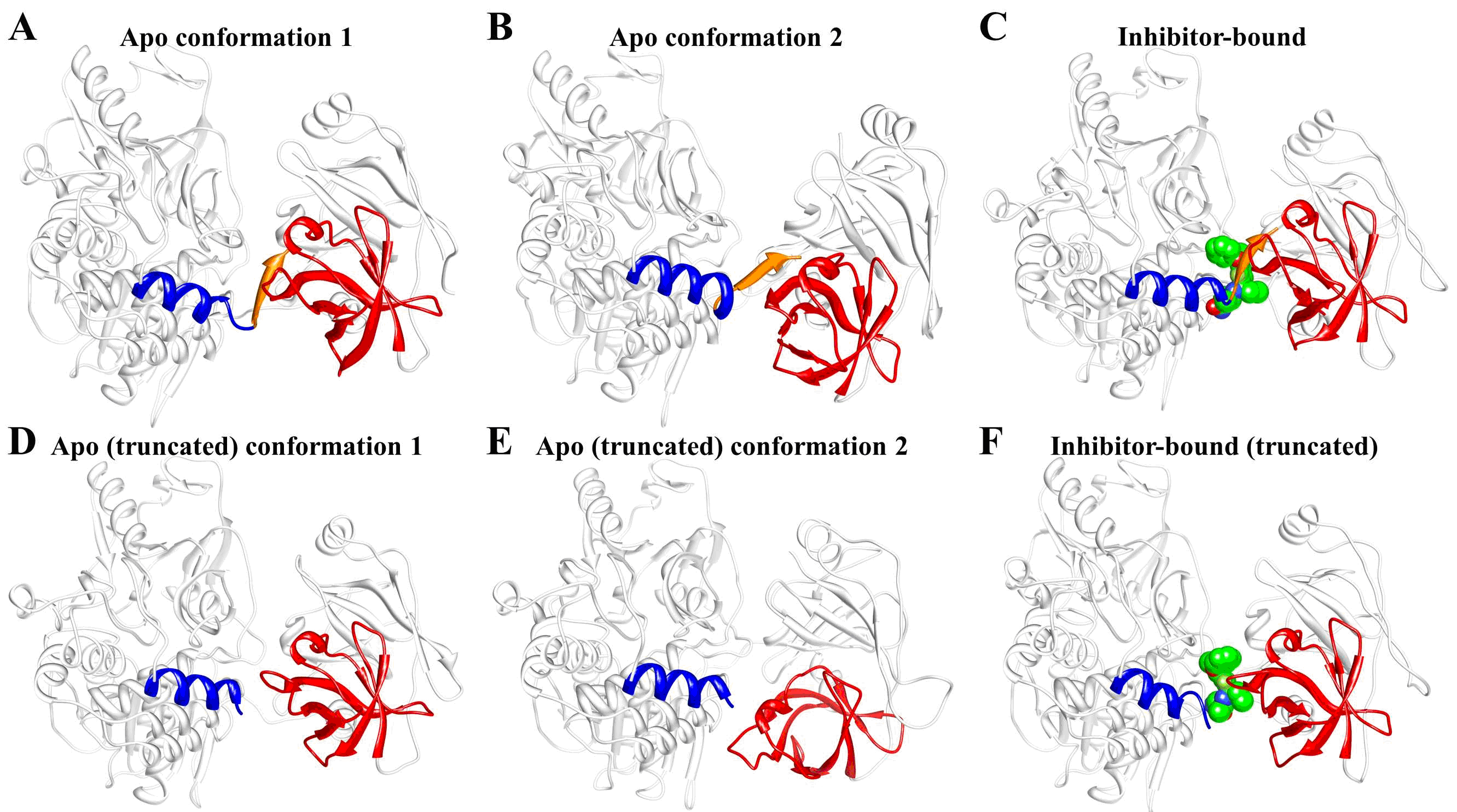

Supplement: Figure S1 — Clustering analysis of HCV NS3/4A protein motions in different models. A–F are the representative structural conformations of generated clusters for apo, inhibitor-bound, apo (truncated), and inhibitor-bound (truncated) HCV NS3/4A protein during the 100 ns of molecular dynamics simulation. The regions of protein are individually colored: the helicase residues 614–625 are shown in blue, the C-terminal portion (amino acids 626–631) of the helicase domain orange, the protease residues 103–171 red, the inhibitor green, and the others protein gray. (TIF) [file pone.0087077.s001.tif]

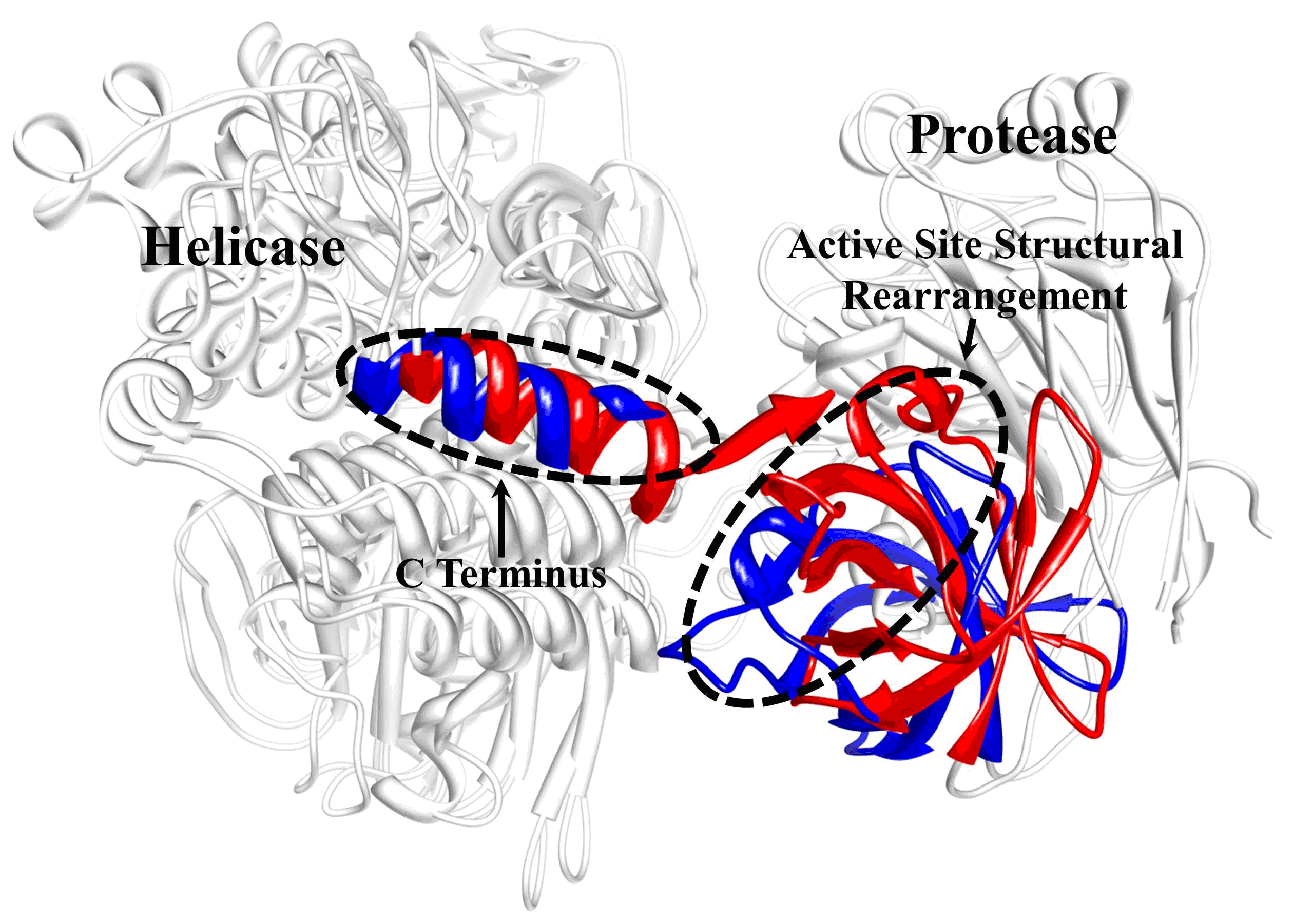

Supplement: Figure S2 — The aligned apo and its truncated form of HCV NS3/4A protein structures. The regions of protein are individually colored: the helicase residues 614–625, the C-terminal portion (amino acids 626–631) of the helicase domain, and the protease residues 103–171 of the apo structure are shown in red, the helicase residues 614–625 and the protease residues 103–171 of the truncated apo structure are shown in blue, and the others protein gray. (TIF) [file pone.0087077.s002.tif]
